# Trends and disparity in the provision and consumption of essential medicines in China from 2016 to 2021: institutional, regional, and economic variations

**DOI:** 10.3389/fpubh.2025.1555598

**Published:** 2025-06-10

**Authors:** Mingyue Zhao, Chenglong Lin, Shengjie Ding, Yubei Han, Yuhan Zhao, Ali Hassen Gillani, Yu Fang

**Affiliations:** ^1^Department of Pharmacy Administration and Clinical Pharmacy, School of Pharmacy, Xi’an Jiaotong University, Xi’an, China; ^2^Center for Drug Safety and Policy Research, Xi’an Jiaotong University, Xi’an, Shaanxi, China; ^3^Shaanxi Centre for Health Reform and Development Research, Xi’an, China

**Keywords:** essential medicines availability, global health, equitable healthcare access, China’s healthcare system, EMs availability and consumption, regional disparities

## Abstract

**Objective:**

Analyze the provision and consumption of essential medicines (EMs) across healthcare institutions, regions, and levels of economic development. Evaluate the mechanisms by which EMs policies promote their own implementation.

**Methods:**

Using national drug utilization monitoring database (2016–2021), we perform a descriptive analysis to explore trends and disparities in EM provision and consumption across three institution levels, four regions (from 30 provinces), and different economic development levels in China. Key metrics include the average number and proportion of provided EMs and the consumption rate per healthcare institution. Utilize a two-way fixed-effects regression model to evaluate the relationship between EMs provision and consumption.

**Findings:**

The average provided number of EMs is much lower than that in the national EMs list. Both provision (number and proportion) and consumption of EMs show institutional and regional disparities. There is a moderately positive correlation between EMs provided proportion and GDP (0.66, *p* < 0.01), while the provided proportion and consumption rate are moderately negatively correlated with GDP (−0.66, *p* < 0.01; −0.64, *p* < 0.01). In highly developed regions, EMs supply is highest but utilization lowest; in underdeveloped regions, provision is least but utilization relatively high.

**Conclusion:**

This study shows disparities in EM provision and consumption across institutions, regions, and economic levels in China. Although essential medicine policy coordination with other policies needs improvement, targeted interventions are needed to bridge gaps in less developed regions and promote medicine equity.

## Introduction

The World Health Organization (WHO) defined essential medicines (EMs) as “those that satisfy the priority health care needs of the population” (1). In 1977, the National EMs List (NEML) was firstly constructed by WHO (2). The Lancet’s commission identified five areas that are crucial to EMs policies: paying for a basket of EMs, making EMs affordable, assuring the quality and safety of medicines, promoting quality use of medicines, and developing missing EMs (3). Sustainable and universal access to EMs has been considered a basic human right, and they ought to be available at all times, in the proper dosage forms, to all segments of society.

China introduced the concept of EMs in the 1980s and established the National Essential Medicines Policy (NEMP) in 2009 as part of the new healthcare reform. Since then, three editions of the NEML have been published in 2009, 2012, and 2018, demonstrating a clear expansion trajectory from an initial 307 drug species to 520, and subsequently to 685 (4–6). China has made efforts to ensure the quality, safety, and affordability of EMs (7, 8). However, shortages and utilization challenges of EMs remain significant issues globally and in China (9–12). Persistent criticism has emerged regarding the lack of synchronized policy frameworks to support these lists updates. Until 2019, the Chinese government launched new support policies under the NEMP, incentivizing government-owned healthcare facilities to use EMs (13). The “986” policy was introduced to set progressive targets for EM provision as well as to enhance the utilization of EMs through their provision: at least 90% (the ratio of EMs to the total number of medicines) in primary healthcare institutions, 80% in secondary public hospitals, and 60% in tertiary public hospitals (14).

Most studies on EMs in China focus on provision and consumption within individual hospitals or regions, without national-scale research, especially following recent policy interventions (15–18). This study investigates trends and variations in EM provision and consumption across healthcare institutions, regions, and economic levels in China and assesses whether policy measures promoting provision enhance EM consumption.

## Methods

### Data sources

This study utilized data from two sources. The first dataset, covering 2016–2021, was obtained from the national drug utilization monitoring database managed by the National Health Commission of China. The database comprises drug provision and consumption data from 47,094 medical institutions nationwide, including 2,927 tertiaries, 7,289 secondary and 36,878 primary hospitals in 2021 (see Supplementary Table S1). The second dataset was sourced from the National Bureau of Statistics of China and the China Health Yearbook.

### Variables

The provision of EMs can be assessed through two key indicators: the total number of EMs (by generic name) and the proportion of EMs provided relative to all medicines in a health institution. The total number reflects EMs diversity, while the proportion indicates the structural representation of provided medicines. Furthermore, the proportion serves as a measure of compliance with the “986” policy, which mandates specific thresholds for EM provision.

The consumption of EMs is quantified by the rate of prescribed EMs, calculated as the total units of prescribed EMs per health institution divided by the total units of prescribed medicines within that institution. Note that the research conducted here does not base on the prescription level. The term “prescribed” mentioned throughout this paper refers to the consumption of EMs. We have not extracted hospital prescriptions for statistical analysis. Instead, our analysis is based on “Medication usage data from public medical institutions,” as obtained from the Drug Use Monitoring Report. The volume of EM consumption measured in units such as ten thousand pieces, units, tablets, or bottles.


$$\text{Average Number of Provided}\;\text{EMs}=\frac{\sum_{i=1}^{N}\;\text{Provided}\;\text{EMs}\;\text{in hospital}\;i}{N}$$

$$\begin{array}{l} \text{Proportion of}\;\\ \text{Provided}\;\text{EMs}=\frac{\sum_{i=1}^{N}(\frac{\text{Provided}\;\text{EMs}\;\text{in hospital}\;i}{\text{Total Provided Medicines in hospital}\;i})}{N} \end{array}$$

$$\begin{array}{l} \text{Average Rate of}\;\\ \text{Prescribed}\;\text{EMs}=\frac{\sum_{i=1}^{N}(\frac{\text{Prescribed}\;\text{EMs}\;\text{in hospital}\;i}{\text{Total Prescribed Medicines in hospital}i})}{N} \end{array}$$

### Statistical analysis

This study hypothesizes that the late-2018 expansion of the NEML and the 2019 implementation of “986” policy exert comparable structural impacts on pharmaceutical supply systems, jointly driving statistically significant post-2019 growth in provision and consumption of EMs in China. We conducted a descriptive analysis to examine the provision and consumption of EMs across time periods, levels of healthcare institutions, regions and economic development within China. For the number of provided EMs, we calculated medians with interquartile ranges. For the proportion of provided EMs and the rate of prescribed EMs, we calculated the mean value and standard deviation (SD). The relationship between the provision/consumption and per capita Gross Domestic Product (GDP per capita) was analyzed using spearman correlation analysis. Healthcare institutions are categorized into three levels: primary hospitals, secondary hospitals, and tertiary hospitals. Based on the characteristics of China’s economic geography (see Supplementary Table S2), the nation is divided into four economic regions: eastern, central, western, and northeastern areas. Further study, we divided the 30 provinces into three sub-groups (High, Middle, Low) based on their GDP levels.

To determine whether the increase in the provision of EMs accounted for their increased consumption, a two-way fixed effects regression model was employed to assess the causal relationship between these two factors. Covariates included in the regression analysis were economic factors, healthcare resources, and socio-demographic factors (definitions are presented in Supplementary Table S3). All analyses were conducted using Stata version 17 (StataCorp LP, College Station, United States).

## Results

### The provision and consumption of EMs from 2016 to 2021

**Figure 1:**
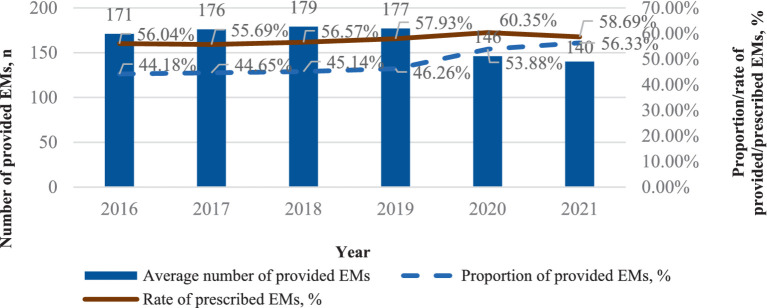
The provision and consumption of EMs per health institute, China, 2016–2021.

As shown in Figure 1, the average median number of provided EMs per health institute is 165 in 30 provinces of China from 2016 to 2021. Between 2016 and 2021, the number of provided EMs increased steadily during the first 4 years but began to decline in 2020, with a decrease of over 18.75% after 2019. Despite this, the proportion of provided EMs rose from 46.26% (in 2019) to 56.33% (in 2021), showing an increase in the first 4 years and a more significant rise after 2019. However, the rate of prescribed EMs grew from 57.93% (in 2019) to 58.69% (in 2021) over the same period.

### Hospital level related provision and consumption of EMs

**Figure 2:**
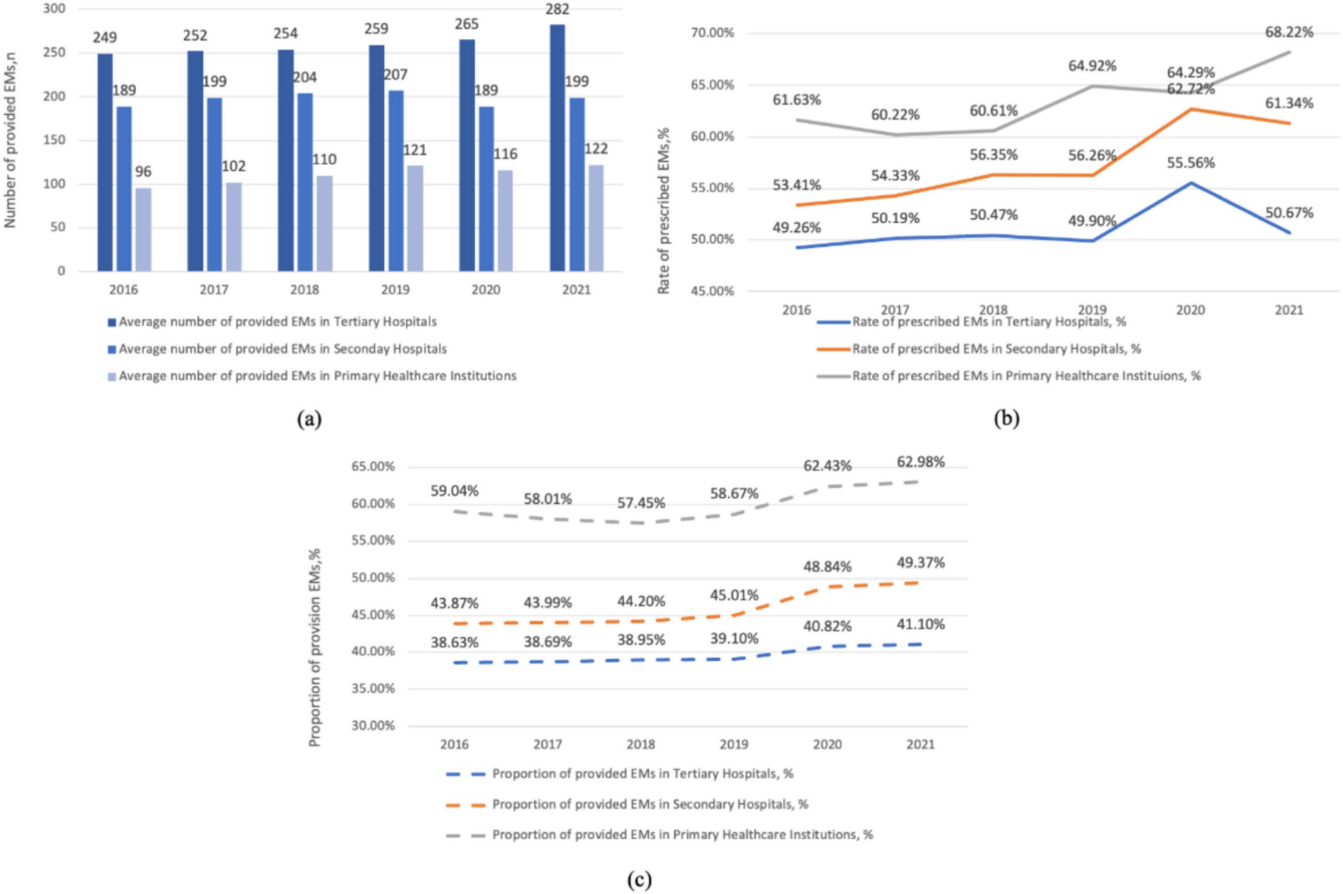
Hospital level related provision and consumption of EMs per health institute, China, 2016–2021. **(a)** The average number of provided EMs in hospitals at various levels. **(b)** The rate of prescribed EMs in hospitals at various levels. **(c)** The proportion of provided EMs in hospitals at various levels.

As depicted in Figure 2, tertiary hospitals boast the largest median number of provided EMs, followed by secondary and primary hospitals. Overall, the number of provided EMs in tertiary and primary hospitals increased slightly (from 249 in 2016 to 282 in 2021 and from 96 in 2016 to 122 in 2021, respectively). However, the increase in provided EMs in secondary hospitals was not as significant (from 189 in 2016 to 199 in 2021). Primary hospitals have the highest proportion of provided EMs, ranging from 57.45 to 62.98%, followed by secondary hospitals (43.87–49.37%) and tertiary hospitals (38.63–41.10%). From 2016 to 2021, the proportion of provided EMs increased in all three levels of healthcare institutions. Moreover, before 2019, the increase was gradual, and after 2019, there was a relatively rapid rise, especially in secondary hospitals and primary hospitals. Tertiary hospitals had the smoothest growth during this period. Primary hospitals have the average highest rate of prescribed EMs (63.32%), followed by secondary (57.40%) and tertiary hospitals (51.01%). The consumption of EMs in tertiary hospitals steadily increased from 2016 to 2020, followed by a slight decrease in 2021. In primary hospitals and secondary hospitals, the consumption of EMs initially rose slowly from 2016 to 2020 but experienced a relatively rapid increase in 2021.

### The provision and consumption of EMs in different regions

**Figure 3:**
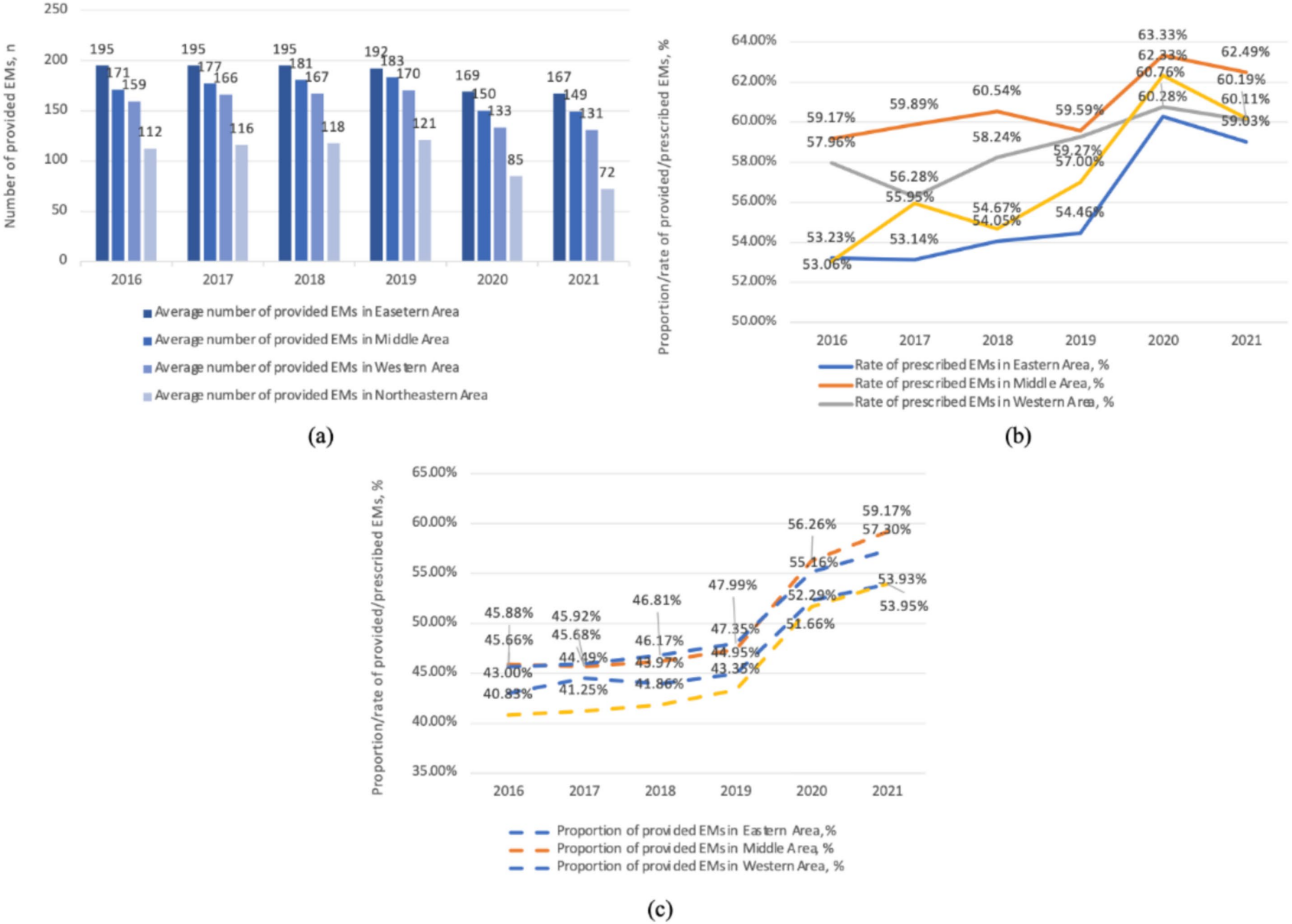
The provision and consumption of EMs in different regions per health institute, China, 2016–2021. **(a)** The average number of provided EMs in various areas. **(b)** The rate of prescribed EMs in various areas. **(c)** The proportion of provided EMs in various areas.

As shown in Figure 3, the average median number of provided EMs is highest in the eastern region (185), followed by the central (168), western (154), and northeastern regions (104), with a noticeable gap among them. The median number of provided EMs in the eastern region is nearly double that of the northeastern region in 2021. Additionally, a clear decline in the number of provided EMs is observed starting in 2020. The proportion of provided EMs is significantly higher in the western (49.81%) and central regions (50.09%) compared to the eastern (47.11%) and northeastern regions (45.48%). This proportion remained stable before 2019, followed by a notable increase across all regions after 2019. Regarding the rate of prescribed EMs, the central region (60.84%) ranks highest, followed by the western (58.77%), northeastern (57.20%), and eastern regions (55.70%). In the northeastern region, there was a rapid increase in the rate of prescribed EMs after 2019, followed by a sharp decrease after 2020 and still be in the penultimate place in 2021.

### The provision and consumption of EMs at different GDP level

**Figure 4:**
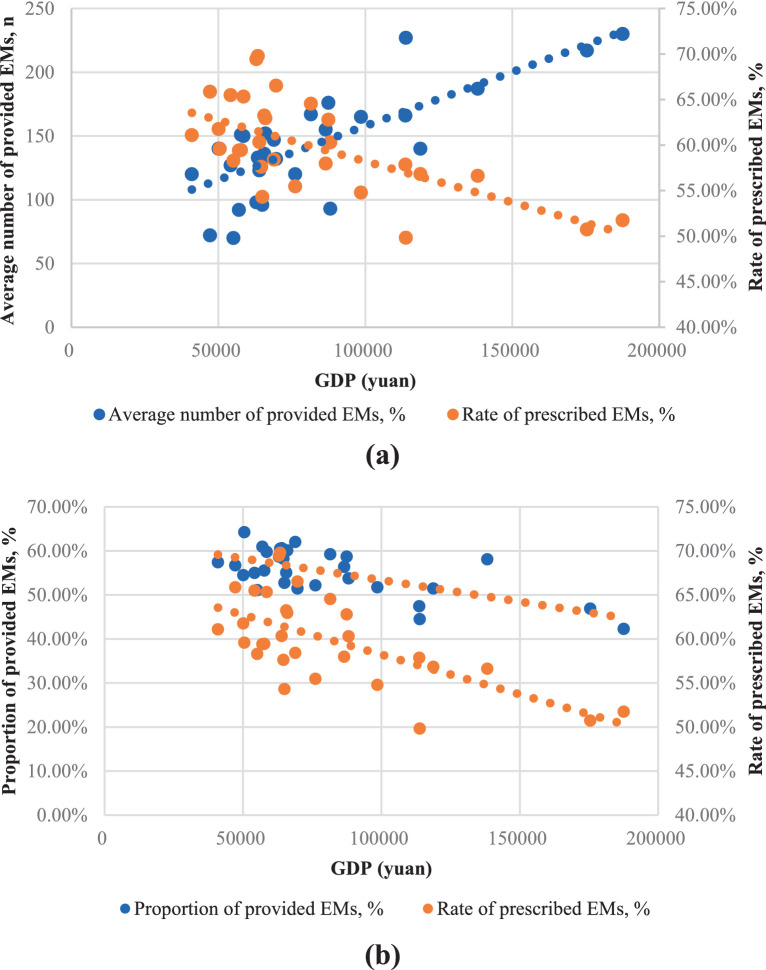
The provision and consumption of EMs per health institute in relation to provincial GDP of China, 2021. **(a)** The horizontal axis was the average number of provided EMs; **(b)** The horizontal axis was the proportion of provided EMs.

Figure 4a underscores the relationship between the number of provided EMs and GDP, with each point representing a province. Apparently, provinces with higher GDP tend to have a larger quantity of provided EMs (0.66, *p* < 0.01). However, Figure 4b shows an inverse trend for the proportion of provided EMs (−0.66, *p* < 0.01) and the consumption rate of EMs (−0.64, *p* < 0.01) in relation to GDP.

**Figure 5:**
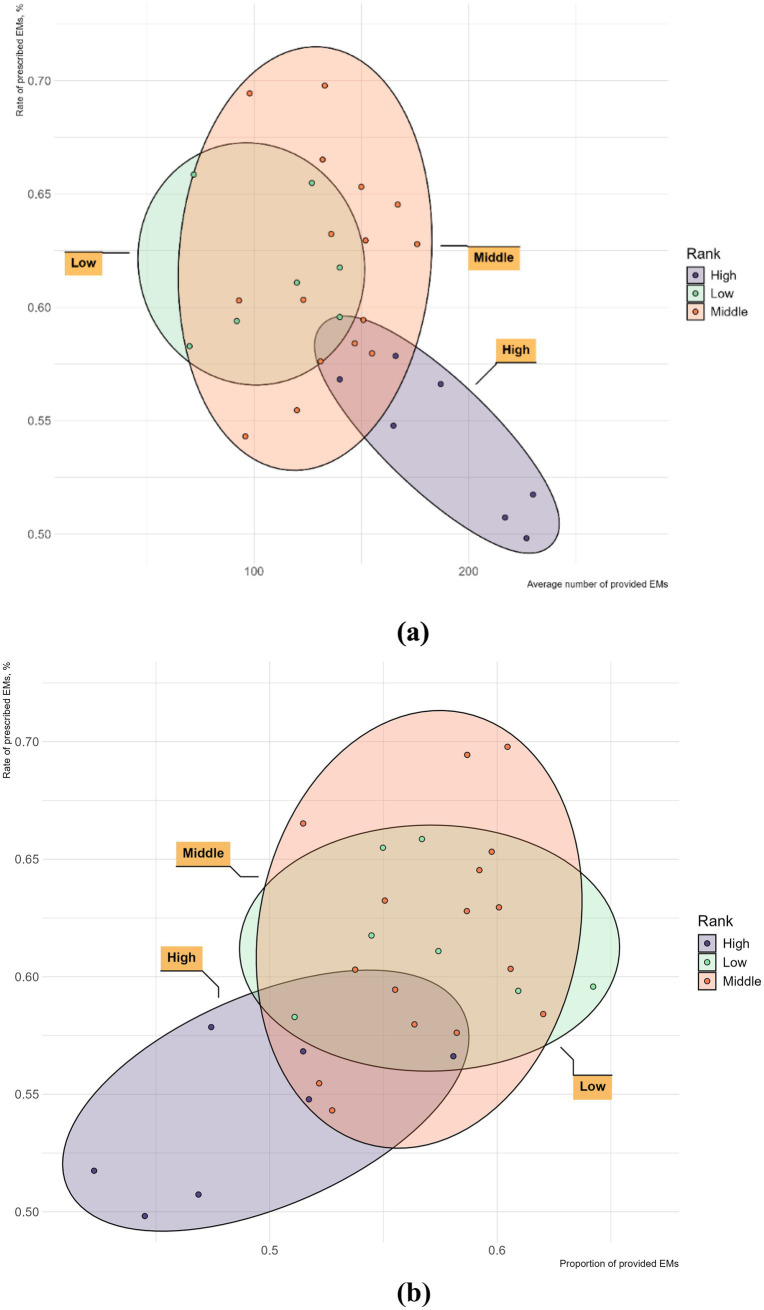
The provision and consumption of EMs per health institute in different GDP group of China, 2021. **(a)** The horizontal axis is the average number of provided EMs; **(b)** The horizontal axis is the proportion of provided EMs.

Further study, Figure 5a explores the relationship between the number of provided EMs and the rate of prescribed EMs within each GDP sub-group. In the high-GDP regions, the rate of prescribed EMs is inversely proportional to the number of provided EMs. In the middle-GDP regions, however, the rate of prescribed EMs is directly proportional to the number of provided EMs. In the low-GDP regions, there appears to be no clear proportional relationship between these two factors. Figure 5b shows a positive trend between the rate of prescribed EMs and the proportion of provided EMs in all GDP regions.

### Causal effects of the provision on the consumption of EMs

Table 1 presents regression results between the proportion of provided EMs and the rate of prescribed EMs. The provision proportion coefficient is significant in all models. Model 1 indicates that the proportion of provided EMs significantly influences the prescribed rate (*R*^2^ = 0.242). Model 2 includes additional covariates for robustness. Models 3 and 4 control for unobservable individual differences and time trends with fixed effects. In Model 5, which controls for both fixed and time effects, the provision coefficient on EMs consumption is 0.464 (*p* < 0.05), and the model explains nearly a quarter of the variation. Thus, it is concluded that the provision proportion of EMs has a significant positive influence on their consumption.

**Table 1:** Regression results of the provision on the consumption of EMs.

Variables	1	2	3	4	5
Provision	0.522^***^	0.474^***^	0.309^***^	0.384^***^	0.464^**^
	(0.084)	(0.098)	(0.061)	(0.145)	(0.189)
Constant	0.335^***^	0.59	0.437^***^	0.76	0.943
	−0.042	−0.385	−0.029	−0.995	−1.286
Control variables	No	Yes	No	Yes	Yes
Fixed effects	No	No	Yes	Yes	Yes
Time effects	No	No	No	No	Yes
*R* ^2^	0.242	0.486	0.147	0.226	0.241

Standard errors in parentheses. ^*^ *p* < 0.1, ^**^ *p* < 0.05, ^***^ *p* < 0.01.

## Discussion

As shown in Figures 1, 3, the number of provided EMs declined after 2019. In contrast, the proportion and number of provided EMs in all three levels of healthcare institutions increased (see Figure 2). The decline from 2016 to 2021 is due to the inclusion of primary hospitals in 2019, as they have fewer provided EMs than hospitals at other levels.

The number of provided EMs varies significantly across different levels of hospitals, reflecting their distinct functions and rankings. However, the number of provided EMs in each level of hospitals remain substantially below the quantity of NEML. Moreover, a review of national and provincial drug shortage lists in China from 2018 to 2021 revealed that 30.4% of EMs were in shortage, accounting for 51.0% of all shortages (19). Additionally, similar shortages of EMs have been frequently reported in various countries across America, Asia, and African (20–22).

The diversity of provided EMs varies across different regions of China. Our findings align with previous studies and reviews showing that EMs provision shows regional disparities (10, 23, 24). The western region, distant from manufacturing centers and seaports and with mountainous terrain, faces transportation challenges. In contrast, the northeastern region with plains and a developed transportation network still has a gap in EMs provision compared to the western region, indicating other factors also influence EMs provision.

Regions with higher GDP levels tend to have a greater number of provided EMs, while in low economic development areas, the supply of EMs appears to be unrelated to GDP. This result highlights the inequality in EMs provision across regions with varying levels of economic development in China. This phenomenon is not exclusive to China. Generally speaking, countries with lower GDP and development levels tend to have shorter NEMLs. However, there are many exceptions (25). This might be because low- and middle-income countries often encounter delays in accessing new EMs (26). In contrast, countries with higher GDPs typically have stronger healthcare systems that are better resourced and more capable of addressing shortages (27–30).

We believe that the increase in the proportion of provided EMs after 2019 is primarily due to the implementation of “986” policy (Figure 1). This policy was also incorporated into the assessment criteria for medical institutions, further encouraging the rise in the proportion of provided EMs. However, our results may be overestimated (as the sample changed after 2019).

However, due to the “986” policy not being mandatory, the proportion of provided EMs still has not reached the policy goal in different levels of hospitals. In fact, the proportion of EMs provision is constrained by multiple policy factors. Firstly, in late 2018, China implemented volume-based procurement policy to promote generic drug substitution (31). Winning drugs gain large market share; unsuccessful ones have smaller share. Generic drugs from this policy are required for use by medical institutions through administrative intervention. As of the eighth batch, 68 pharmacological categories cover 63% of EMs list (32). Secondly, as brand drug substitutes, generic drugs must pass quality and efficacy consistency evaluation. By May 2023, evaluated generics account for only 43% of EML (33). Thirdly, in China’s public medical institutions, the quantity of drugs supplied by hospitals is restricted by hospital drug formulary, indirectly limiting drug provision (34).

As shown in Figure 3, the proportion of provided EMs varies by regions. The proportion of provided EMs is negatively correlated with GDP (Figure 4b). In central and western regions, which are less economically developed areas, the proportion of EMs provision is comparatively favorable. While northeast (the least developed regions) has a lower proportion of provided EMs, but for different reasons. Its hospitals have a low total drug allocation and the lowest number of provided EMs. The eastern region has the lowest proportion of provided EMs due to more non-EMs.

For the consumption rate of EMs, Figure 1 shows an increase in 2021 compared to 2016, but a decrease compared to 2020. Coupled with the rise in EM consumption in primary hospitals after 2019, as seen in Figure 2, we believe that the increase in EM consumption after 2019 in Figure 1 is not solely due to policy changes. It may also be attributed to statistical reasons (mentioned above).

In Figure 2, the rate of prescribed EMs in tertiary hospitals decreased in 2021 compared to 2020, which is the primary reason for the overall decrease in consumption shown in Figure 1. We believe this is because the “986” policy was not mandatory for health institutions, whereas the volume-based purchasing policy was compulsory, leading to an increased use of volume-based purchasing medicines in health institutions. We can see that the consumption of EMs in primary healthcare centers are more than that of the secondary and tertiary healthcare centers.

As shown in Figure 3, the middle and western regions have higher levels of EM consumption which may due to the higher proportion of provided EMs. In the northeastern region, the lower EM consumption may be a result of the lowest proportion of provided EMs as well as lowest level of provided EMs number. In contrast, the eastern region, with the highest GDP development, offers more options beyond EMs, leading to a lower rate of prescribed EMs. However, the eastern region owns the largest number of EM provision. As shown in Figure 4, EMs consumption has a negative linear relation with GDP. However, at lower level of GDP development provinces, the relationship again is quite scattering.

In terms of the relationship between provision and consumption of EMs, on the one hand, we found that more developed economy leads to better EMs provided diversity but also more alternative drugs, resulting in lower EMs utilization rate. Hence, in highly developed regions, although the diversity of EMs allocation requires enhancement, what demands even greater improvement is the active utilization rate of EMs. In middle-GDP regions, the positive correlation between the number of provided EMs and their consumption suggests that increasing EMs provision could lead to higher EMs consumption. However, in low-GDP regions, the data is more scattered with no obvious pattern, and the reasons are even more complex. For example, in Xinjiang (XJ) strongly implemented the “986” policy, leading to high EM consumption despite its low GDP (35). Conversely, the target for promoting EMs in Jilin (JL) under the “986” policy was not as ambitious as that of Heilongjiang (HL), resulting in a lower consumption rate (36). Provinces like Yunnan (YN), Guizhou (GZ), Guangxi (GX), and Gansu (GS) show relatively high numbers of provided EMs but low consumption, likely due to the prevalent use of regional traditional herbal medicines (37). On the other hand, it is evident that increasing the proportion of provided EMs tends to promote EMs consumption across all regions. Our regression results also show that the proportion of provided EMs is positively correlated with consumption rate, in line with a Chinese hospital data study (38). This confirms that the proportion of provided EMs is a key factor in the success of the “986” policy, making its focus on increasing EMs provision reasonable.

Our study has limitations. First, four regions (Tibet, Taiwan, Hong Kong, and Macau) were excluded due to unavailable data. The study covered 30 provinces in China, encompassing all secondary and tertiary medical institutions. Second, a large number of primary medical institutions were included in the sample after 2019, which led to changes in statistical indicators. Finally, this study is grounded in macro-level data. Due to the absence of individual data, it cannot be extended to the per capita level.

## Conclusion

In conclusion, this study reveals a significant upward trend in the provision and consumption of EMs, while also uncovering variations across different institutions and regions. These disparities underscore the impact of socio-economic factors on healthcare accessibility and the use of EMs. It should be noted that China’s highly imbalanced socio-economic development results in inequality in the provision and utilization of EMs. Although the objective of achieving a certain proportion of provided EMs under the “986 policy” could potentially enhance the consumption of EMs, it is constrained by volume-based purchasing, quality-efficiency consistency evaluation, and limitations on hospital formularies. Consequently, the synergy between the National Essential Medicines Policy (NEMP) and other policies has not been fully achieved in China.

## Data Availability

The original contributions presented in the study are included in the article/Supplementary material, further inquiries can be directed to the corresponding author.
