# Supplementary material for: Trends and disparity in the provision and consumption of essential medicines in China from 2016 to 2021: institutional, regional, and economic variations
Source: Front Public Health. 2025 Jun 10;13:1555598. doi: 10.3389/fpubh.2025.1555598 (PMC12185506; doi:10.3389/fpubh.2025.1555598)
Supplement: Supplementary file 1 [file Table_1.DOCX]

**Supplementary Table S1. Information on the Number of Sample Hospitals in China, 2016 -2021**

| **Year** | **Tertiary Hospital** | **Secondary Hospital** | **Primary Healthcare Institution** | **Total** |
| --- | --- | --- | --- | --- |
| **2016** | 2084 | 2924 | 3111 | 8119 |
| **2017** | 2159 | 3121 | 3650 | 8930 |
| **2018** | 2225 | 3357 | 4227 | 9809 |
| **2019** | 2287 | 3674 | 5175 | 11136 |
| **2020** | 2664 | 6745 | 24386 | 33795 |
| **2021** | 2927 | 7289 | 36878 | 47094 |

**Supplementary Table S2. Socio-economics characteristics, by area, China, 2016-2021**

| **Characteristics^a^** | **Year** | | | | | |
| --- | --- | --- | --- | --- | --- | --- |
|  | **2016** | **2017** | **2018** | **2019** | **2020** | **2021** |
| **National** |  |  |  |  |  |  |
| Population in 10,000 | 138663 | 139430 | 139956 | 140444 | 140647 | 140694 |
| GDP per capita (CHY) | 54071.79 | 59581.68 | 65204.00 | 69823.04 | 71671.26 | 81055.54 |
| **Eastern^b^** |  |  |  |  |  |  |
| Population in 10,000 | 54787 | 55283 | 55706 | 56074 | 56435 | 56605 |
| GDP per capita (CHY) | 72299.94 | 79079.10 | 85516.52 | 90910.30 | 93124.80 | 105269.69 |
| **Middle^b^** |  |  |  |  |  |  |
| Population in 10,000 | 36331 | 36444 | 36507 | 36573 | 36445 | 36445 |
| GDP per capita (CHY) | 44341.88 | 49462.00 | 55050.57 | 59474.28 | 60498.18 | 68360.41 |
| **Western^b^** |  |  |  |  |  |  |
| Population in 10,000 | 37188 | 37466 | 37641 | 37817 | 37942 | 37915 |
| GDP per capita (CHY) | 40296.04 | 44943.98 | 49841.10 | 53735.22 | 55637.18 | 63244.81 |
| **Northeastern^b^** |  |  |  |  |  |  |
| Population in 10,000 | 10357 | 10237 | 10102 | 9980 | 9825 | 9729 |
| GDP per capita (CHY) | 41242.16 | 43887.86 | 47130.07 | 50226.95 | 51807.43 | 57139.99 |

^a^ Data source: National Bureau of Statistics of China.

^b^ Provinces included in the eastern region are: Beijing, Tianjin, Hebei, Shanghai, Jiangsu, Zhejiang, Fujian, Shandong, Guangdong, Hainan; in the central region are: Shanxi, Anhui, Jiangxi, Henan, Hubei, Hunan; in the western region are: Inner Mongolia, Guangxi, Chongqing, Sichuan, Guizhou, Yunnan, Shaanxi, Gansu, Qinghai, Ningxia, Xinjiang; in the northeastern region are: Liaoning, Jilin, Heilongjiang.

**Supplementary Table S3. Definition of Control Variables**

| **Theme** | **Variable** | **Definition** |
| --- | --- | --- |
| Financial Factors | GDP | Per capita Gross Domestic Production |
|  | Governance | Local financial medical and health expenditure proportion |
| Healthcare Resources | Patient visits | The daily average number of diagnosis and treatment per doctor |
|  | Inpatients | The average inpatients of the doctor is responsible for the inpatient bed per day |
|  | Primary Healthcare Centers | Proportion of primary healthcare centers |
|  | Tertiary Public Hospitals | Number of tertiary public hospitals per 10,000 population |
|  | Second Public Hospitals | Number of tertiary public hospitals per 10,000 population |
|  | Healthcare Professionals | The number of healthcare technicians per 10,000 population |
|  | Beds in Healthcare Institutions | Number of beds in medical institutions per 10,000 population |
| Sociodemographic | Urban | The proportion of urban population to the permanent population at the end of the year |
|  | Old-age Dependency Ratio | The ratio of the population of the elderly (> 65 years) in the population to the population of labor age |
|  | Youth Dependency Ratio | The ratio of the population of children (0 - 14 years) in a population to the population of labor age |
|  | Gender | Gender Ratio |
|  | Literacy | The proportion of the population of the literacy is 15 years old and above |
|  | Income | Per capita disposable income |
